# (8-Amino­quinoline-κ^2^ *N*,*N*′)bis­(1,1,1,5,5,5-hexa­fluoro­pentane-2,4-dionato-κ^2^ *O*,*O*′)cobalt(II)

**DOI:** 10.1107/S1600536812011312

**Published:** 2012-03-21

**Authors:** David J. Harding, Darunee Sertphon, Phimphaka Harding

**Affiliations:** a Molecular Technology Research Unit, Department of Chemistry, Walailak University, Thasala, Nakhon Si Thammarat 80161, Thailand

## Abstract

In the title compound, [Co(C_5_HF_6_O_2_)_2_(C_9_H_8_N_2_)], the Co^II^ centre exhibits a pseudooctahedral coordination geometry, comprising two N-atom donors from the bidentate amino­quinoline ligand and four O-atom donor atoms from two bidentate chelating 1,1,1,5,5,5-hexafluoropentane-2,4-dionate ligands. In the crystal, molecules are linked *via* pairs of N—H⋯O hydrogen bonds, forming inversion dimers. These dimers are further connected through π–π interactions between neighbouring quinoline rings [centroid–centroid distance = 3.472 (2) Å], and stack along the *c* axis.

## Related literature

For related structures, see: Sertphon *et al.* (2011 ▶); Aakeröy *et al.* (2004 ▶, 2005 ▶, 2007 ▶); Harding *et al.* (2009 ▶, 2010 ▶).
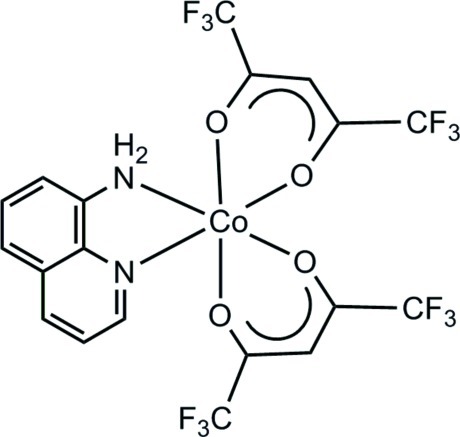


## Experimental

### 

#### Crystal data

[Co(C_5_HF_6_O_2_)_2_(C_9_H_8_N_2_)]
*M* *_r_* = 617.22
Triclinic, 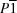

*a* = 9.6102 (4) Å
*b* = 10.2681 (5) Å
*c* = 12.4154 (6) Å
α = 114.149 (1)°
β = 90.927 (1)°
γ = 95.202 (1)°
*V* = 1111.40 (9) Å^3^
*Z* = 2
Mo *K*α radiation
μ = 0.90 mm^−1^
*T* = 100 K
0.29 × 0.18 × 0.16 mm

#### Data collection

Bruker APEXII CCD area-detector diffractometer
Absorption correction: multi-scan (*SADABS*; Bruker, 2003 ▶) *T*_min_ = 0.682, *T*_max_ = 0.746
10217 measured reflections
5113 independent reflections
4735 reflections with *I* > 2σ(*I*)
*R*_int_ = 0.010

#### Refinement

*R*[*F* ^2^ > 2σ(*F* ^2^)] = 0.031
*wR*(*F* ^2^) = 0.078
*S* = 1.05
5113 reflections
399 parameters
54 restraints
H-atom parameters constrained
Δρ_max_ = 0.79 e Å^−3^
Δρ_min_ = −0.55 e Å^−3^

### 

Data collection: *APEX2* (Bruker, 2005 ▶); cell refinement: *APEX2*; data reduction: *SAINT* (Bruker, 2003 ▶); program(s) used to solve structure: *SHELXS97* (Sheldrick, 2008 ▶); program(s) used to refine structure: *SHELXL97* (Sheldrick, 2008 ▶); molecular graphics: *X-SEED* (Barbour, 2001 ▶); software used to prepare material for publication: *publCIF* (Westrip, 2010 ▶).

## Supplementary Material

Crystal structure: contains datablock(s) I, global. DOI: 10.1107/S1600536812011312/bg2451sup1.cif

Structure factors: contains datablock(s) I. DOI: 10.1107/S1600536812011312/bg2451Isup2.hkl

Additional supplementary materials:  crystallographic information; 3D view; checkCIF report

## Figures and Tables

**Table 1:** Hydrogen-bond geometry (Å, °)

*D*—H⋯*A*	*D*—H	H⋯*A*	*D*⋯*A*	*D*—H⋯*A*
N2—H2*A*⋯O2^i^	0.90	2.14	3.024 (2)	169
N2—H2*B*⋯O4^i^	0.90	2.56	3.069 (2)	117

Symmetry code: (i) 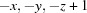.

**Fig. 1.**
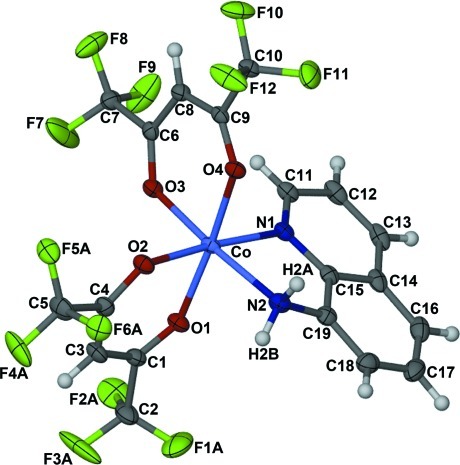
The molecular structure of (1) showing the atom-labelling scheme. Displacement ellipsoids are drawn at the 50% probability level.

**Fig. 2.**
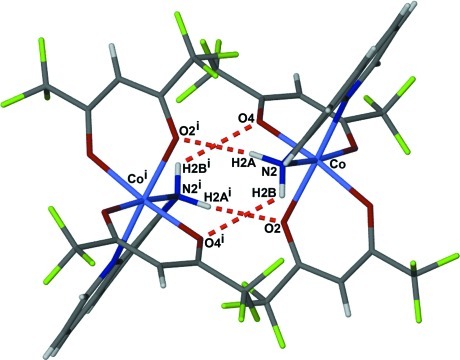
The molecular packing in (1) showing the N—H···O hydrogen bonding interactions forming dimers. Only selected atoms are labelled for clairty. [Symmetry codes: (i) -x, -y, 1 - z].

**Fig. 3.**
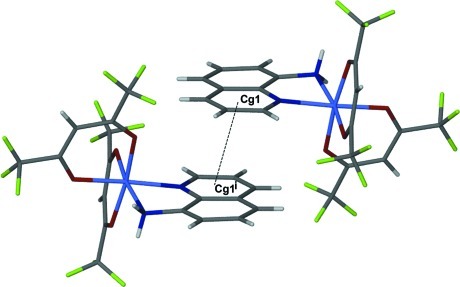
The molecular packing in (1) showing the π···π interactions between the quinolyl ring. Only selected atoms are labelled for clairty. [Symmetry codes: (i) -x, 1 - y, 2 - z].

## supplementary crystallographic information

### Comment

Metal β-diketonates can serve as excellent building blocks in the formation of supramolecular networks. For instance, the use of hydrogen-bonding ligands allows isolation of linear chains (Aakeröy *et al.*, 2004,2005). In this paper we report the synthesis and structure of [Co(hfac)_2_(NH_2_-quin)] (hfac = 1,1,1,5,5,5-hexafluoropentane-2,4-dionato; NH_2_-quin = 8-aminoquinoline).

The reaction of [Co(hfac)_2_(H_2_O)_2_] with 8-aminoquinoline in THF yields [Co(hfac)_2_(NH_2_-quin)] 1 which crystallizes from CH_2_Cl_2_/hexanes in the space group *P*1 (Figure 1). The cobalt metal centre is six-coordinate with a distorted octahedral geometry, the hfac ligands adopting a *cis* arrangement enforced by the chelating NH_2_-quin ligand. The compound is isostructural with [Ni(hfac)_2_(NH_2_-quin)] (Sertphon *et al.*, 2011). The Co—N and Co—O bond lengths (Table 1) are comparable with those reported in *trans*-[*M*(hfac)_2_(py-CH=CH—C_6_F_4_Br)_2_] (Aakeröy *et al.*, 2007) and [Co(hfac)_2_(ppa^Br^)] {ppa^Br^ = 4-bromophenyl(2-pyridylmethylidene)amine, Harding *et al.*, 2010}. The β-diketonate ligands exhibit a *planar* coordination mode in which the angles between the planes defined by the Co and oxygen atoms and the carbon and oxygen atoms of the β-diketonate ligand are 1.7° and 7.6°. Similarly, in *trans*-[*M*(hfac)_2_(py-CH=CH—C_6_F_4_Br)_2_] (*M* = Co, Cu) the β-diketonate ligands are also *planar* (Aakeröy *et al.*, 2007).

The packing in the structure involves two sets of N–H···O interactions between the amino protons of the NH_2_-quin ligand and the coordinated O atoms of the β-diketonate forming discrete dimers {N2—H2A···O2 = 2.135 (2) Å, N2—H2B···O2 2.557 (2) Å, Figure 2 & Table 2}. Similar interactions are also found in [Ni(dbm)_2_(dmae)] (dmae = dimethylaminoethylamine) and appear to be a feature of these types of compounds (Harding *et al.*, 2009). In addition, there is a π-π interaction between the quinolyl rings of neighbouring NH_2_-quin ligands as shown in Figure 3 {*Cg*1—*Cg*1^i^ = 3.472 (2) Å where *Cg*1 is the centroid of the ring C11—C15,N1; i = symmetry code = -*x*, 1 - *y*, 2 - *z*}. The hydrogen bonds mentioned above combine with the π-π interactions to form one-dimensional chains.

### Experimental

To a deep orange red solution of [Co(hfac)_2_(H_2_O)_2_] (1.0300 g, 2 mmol) in THF (10 ml) was added 8-aminoquinoline (0.2884 g, 2 mmol) giving a red-brown solution which was stirred for 2 hr. After evaporating to low volume (*ca*. 2 ml), hexane (3 ml) was added giving an orange precipitate which was filtered and washed with hexanes (2×4 ml) and air dried giving orange microcrystals, 0.8794 g (71%). X-ray quality crystals were grown by layering a CH_2_Cl_2_ solution with hexane (10 ml) leading to orange crystals after 2 days. IR in KBr disc ν_C=O_ 1642 cm^-1^. UV-Vis (in CH_2_Cl_2_, ε mol.dm^-3^cm^-1^) 406sh (340), 416sh (300), 505 (120). C_19_H_10_Co F_12_N_2_O_4_; calc. C 37.0, H 1.6, N 4.5; found C 36.8, H 1.5, N 4.3.

### Refinement

Hydrogen atoms were placed geometrically and refined with a riding model and with *U*_iso_ constrained to be 1.2 (aromatic CH) or 1.5 (NH_2_) × *U*_eq_ of the carrier atom.

Two of the CF_3_ groups in one of the hfac ligands were found to be disordered and were modeled by refining the fluorine atoms in two positions. SIMU and DELU restrainsts were applied resulting in an occupancy of 70/30 (2) for F1A—F3B and 57/43 (3) for F4A—F6B.

### Crystal data

**Table:**

[Co(C_5_HF_6_O_2_)_2_(C_9_H_8_N_2_)]	*Z* = 2
*M**_r_* = 617.22	*F*(000) = 610
Triclinic, *P*1	*D*_x_ = 1.844 Mg m^−^^3^
Hall symbol: -P 1	Mo *K*α radiation, λ = 0.71073 Å
*a* = 9.6102 (4) Å	Cell parameters from 6750 reflections
*b* = 10.2681 (5) Å	θ = 2.7–27.6°
*c* = 12.4154 (6) Å	µ = 0.90 mm^−^^1^
α = 114.149 (1)°	*T* = 100 K
β = 90.927 (1)°	Prism, orange
γ = 95.202 (1)°	0.29 × 0.18 × 0.16 mm
*V* = 1111.40 (9) Å^3^	

### Data collection

**Table:**

Bruker APEXII CCD area-detector diffractometer	5113 independent reflections
Radiation source: fine-focus sealed tube	4735 reflections with *I* > 2σ(*I*)
Graphite monochromator	*R*_int_ = 0.010
φ and ω scans	θ_max_ = 27.6°, θ_min_ = 2.1°
Absorption correction: multi-scan (*SADABS*; Bruker, 2003)	*h* = −12→12
*T*_min_ = 0.682, *T*_max_ = 0.746	*k* = −13→13
10217 measured reflections	*l* = −15→16

### Refinement

**Table:**

Refinement on *F*^2^	Primary atom site location: structure-invariant direct methods
Least-squares matrix: full	Secondary atom site location: difference Fourier map
*R*[*F*^2^ > 2σ(*F*^2^)] = 0.031	Hydrogen site location: inferred from neighbouring sites
*wR*(*F*^2^) = 0.078	H-atom parameters constrained
*S* = 1.05	*w* = 1/[σ^2^(*F*_o_^2^) + (0.0369*P*)^2^ + 0.8282*P*] where *P* = (*F*_o_^2^ + 2*F*_c_^2^)/3
5113 reflections	(Δ/σ)_max_ = 0.001
399 parameters	Δρ_max_ = 0.79 e Å^−^^3^
54 restraints	Δρ_min_ = −0.55 e Å^−^^3^

### Special details

**Table:**

Geometry. All e.s.d.'s (except the e.s.d. in the dihedral angle between two l.s. planes) are estimated using the full covariance matrix. The cell e.s.d.'s are taken into account individually in the estimation of e.s.d.'s in distances, angles and torsion angles; correlations between e.s.d.'s in cell parameters are only used when they are defined by crystal symmetry. An approximate (isotropic) treatment of cell e.s.d.'s is used for estimating e.s.d.'s involving l.s. planes.
Refinement. Refinement of *F*^2^ against ALL reflections. The weighted *R*-factor *wR* and goodness of fit *S* are based on *F*^2^, conventional *R*-factors *R* are based on *F*, with *F* set to zero for negative *F*^2^. The threshold expression of *F*^2^ > σ(*F*^2^) is used only for calculating *R*-factors(gt) *etc*. and is not relevant to the choice of reflections for refinement. *R*-factors based on *F*^2^ are statistically about twice as large as those based on *F*, and *R*- factors based on ALL data will be even larger.

### Fractional atomic coordinates and isotropic or equivalent isotropic displacement parameters (Å^2^)

**Table:**

	*x*	*y*	*z*	*U*_iso_*/*U*_eq_	Occ. (<1)
Co1	0.12629 (2)	0.20101 (2)	0.679585 (17)	0.01788 (7)	
O1	0.17857 (12)	0.15131 (12)	0.82150 (10)	0.0233 (2)	
O2	0.23066 (12)	0.02680 (12)	0.57507 (10)	0.0226 (2)	
O3	0.30314 (12)	0.34361 (12)	0.71520 (10)	0.0220 (2)	
O4	0.08649 (12)	0.23137 (11)	0.52671 (9)	0.0206 (2)	
N1	0.00333 (14)	0.36365 (14)	0.78304 (11)	0.0194 (3)	
N2	−0.07051 (15)	0.07814 (14)	0.66194 (12)	0.0228 (3)	
H2A	−0.1131	0.0575	0.5909	0.027*	
H2B	−0.0573	−0.0051	0.6667	0.027*	
C18	−0.27315 (19)	0.0996 (2)	0.78771 (16)	0.0305 (4)	
H18	−0.2966	0.0007	0.7528	0.037*	
C8	0.26994 (16)	0.41960 (17)	0.55914 (14)	0.0210 (3)	
H8	0.3061	0.4845	0.5294	0.025*	
C13	−0.16746 (19)	0.5479 (2)	0.94323 (14)	0.0289 (4)	
H13	−0.2230	0.6101	0.9972	0.035*	
C6	0.33529 (16)	0.41998 (16)	0.66082 (13)	0.0195 (3)	
C1	0.24544 (17)	0.05082 (17)	0.81746 (14)	0.0228 (3)	
C9	0.15234 (16)	0.32484 (16)	0.50105 (13)	0.0187 (3)	
C3	0.30849 (18)	−0.04772 (18)	0.72205 (15)	0.0262 (3)	
H3	0.3610	−0.1127	0.7344	0.031*	
C19	−0.15744 (17)	0.15903 (18)	0.75482 (14)	0.0225 (3)	
C10	0.09259 (17)	0.33064 (17)	0.38720 (14)	0.0224 (3)	
C11	0.03646 (18)	0.50439 (17)	0.83206 (14)	0.0232 (3)	
H11	0.1197	0.5415	0.8131	0.028*	
C4	0.29504 (17)	−0.05146 (17)	0.60907 (14)	0.0225 (3)	
C14	−0.20721 (17)	0.39818 (19)	0.89415 (14)	0.0250 (3)	
C7	0.46039 (17)	0.53403 (19)	0.71976 (15)	0.0250 (3)	
C2	0.2559 (2)	0.0349 (2)	0.93543 (17)	0.0350 (4)	
C12	−0.04797 (19)	0.60071 (18)	0.91146 (15)	0.0279 (4)	
H12	−0.0223	0.6992	0.9418	0.033*	
C16	−0.32669 (19)	0.3335 (2)	0.92501 (16)	0.0319 (4)	
H16	−0.3849	0.3903	0.9800	0.038*	
C17	−0.35721 (19)	0.1878 (2)	0.87441 (17)	0.0349 (4)	
H17	−0.4344	0.1461	0.8974	0.042*	
C5	0.3599 (2)	−0.1715 (2)	0.50891 (17)	0.0334 (4)	
C15	−0.11961 (16)	0.30965 (17)	0.81087 (13)	0.0206 (3)	
F1A	0.1331 (3)	0.0212 (7)	0.9737 (4)	0.0637 (14)	0.70 (2)
F2A	0.3201 (4)	0.1603 (4)	1.0201 (3)	0.0529 (8)	0.70 (2)
F3A	0.3336 (7)	−0.0600 (6)	0.9363 (4)	0.0808 (18)	0.70 (2)
F1B	0.232 (2)	0.1358 (14)	1.0254 (6)	0.114 (7)	0.30 (2)
F2B	0.3649 (9)	−0.0234 (15)	0.9466 (11)	0.076 (4)	0.30 (2)
F3B	0.1601 (11)	−0.0845 (17)	0.9226 (9)	0.086 (5)	0.30 (2)
F4A	0.4341 (13)	−0.2489 (11)	0.5421 (3)	0.050 (2)	0.57 (3)
F5A	0.4082 (15)	−0.1279 (15)	0.4265 (11)	0.0323 (15)	0.57 (3)
F6A	0.2463 (6)	−0.2711 (5)	0.4388 (4)	0.0334 (10)	0.57 (3)
F4B	0.4928 (14)	−0.1892 (18)	0.5544 (7)	0.048 (3)	0.43 (3)
F5B	0.426 (2)	−0.1294 (19)	0.4389 (17)	0.042 (3)	0.43 (3)
F6B	0.2903 (18)	−0.2858 (7)	0.4676 (15)	0.061 (4)	0.43 (3)
F7	0.55505 (16)	0.48660 (17)	0.76660 (18)	0.0789 (6)	
F8	0.52320 (13)	0.58172 (16)	0.64755 (11)	0.0496 (4)	
F9	0.41614 (15)	0.64738 (15)	0.80499 (13)	0.0618 (4)	
F10	0.15304 (14)	0.43584 (13)	0.36377 (11)	0.0434 (3)	
F11	−0.04305 (12)	0.34529 (16)	0.39296 (11)	0.0457 (3)	
F12	0.10584 (16)	0.20821 (13)	0.29438 (10)	0.0489 (3)	

### Atomic displacement parameters (Å^2^)

**Table:**

	*U*^11^	*U*^22^	*U*^33^	*U*^12^	*U*^13^	*U*^23^
Co1	0.02444 (12)	0.01408 (11)	0.01511 (11)	0.00426 (8)	0.00061 (7)	0.00557 (8)
O1	0.0310 (6)	0.0218 (5)	0.0190 (5)	0.0071 (5)	0.0003 (4)	0.0095 (4)
O2	0.0300 (6)	0.0178 (5)	0.0196 (5)	0.0071 (4)	0.0017 (4)	0.0064 (4)
O3	0.0248 (5)	0.0212 (5)	0.0212 (5)	0.0030 (4)	−0.0018 (4)	0.0100 (4)
O4	0.0269 (6)	0.0169 (5)	0.0177 (5)	0.0031 (4)	−0.0005 (4)	0.0068 (4)
N1	0.0253 (6)	0.0176 (6)	0.0142 (6)	0.0049 (5)	−0.0014 (5)	0.0049 (5)
N2	0.0294 (7)	0.0173 (6)	0.0196 (6)	0.0025 (5)	−0.0008 (5)	0.0055 (5)
C18	0.0303 (9)	0.0332 (9)	0.0310 (9)	−0.0003 (7)	−0.0017 (7)	0.0172 (8)
C8	0.0225 (7)	0.0219 (7)	0.0210 (7)	0.0029 (6)	0.0012 (6)	0.0110 (6)
C13	0.0335 (9)	0.0317 (9)	0.0170 (7)	0.0169 (7)	−0.0017 (6)	0.0030 (6)
C6	0.0191 (7)	0.0189 (7)	0.0203 (7)	0.0062 (5)	0.0025 (5)	0.0070 (6)
C1	0.0257 (8)	0.0223 (7)	0.0232 (8)	0.0021 (6)	−0.0014 (6)	0.0125 (6)
C9	0.0228 (7)	0.0177 (7)	0.0168 (7)	0.0076 (6)	0.0020 (5)	0.0070 (6)
C3	0.0313 (8)	0.0231 (8)	0.0277 (8)	0.0100 (6)	0.0005 (7)	0.0125 (7)
C19	0.0253 (8)	0.0244 (8)	0.0179 (7)	0.0023 (6)	−0.0027 (6)	0.0090 (6)
C10	0.0280 (8)	0.0194 (7)	0.0205 (7)	0.0019 (6)	−0.0027 (6)	0.0091 (6)
C11	0.0295 (8)	0.0186 (7)	0.0196 (7)	0.0046 (6)	−0.0033 (6)	0.0057 (6)
C4	0.0250 (8)	0.0170 (7)	0.0244 (8)	0.0042 (6)	0.0012 (6)	0.0071 (6)
C14	0.0267 (8)	0.0329 (9)	0.0159 (7)	0.0110 (7)	−0.0016 (6)	0.0090 (6)
C7	0.0239 (8)	0.0281 (8)	0.0244 (8)	0.0001 (6)	−0.0025 (6)	0.0129 (7)
C2	0.0400 (10)	0.0456 (11)	0.0310 (9)	0.0172 (9)	0.0071 (8)	0.0246 (9)
C12	0.0376 (9)	0.0198 (8)	0.0211 (8)	0.0104 (7)	−0.0054 (7)	0.0020 (6)
C16	0.0266 (8)	0.0496 (11)	0.0229 (8)	0.0130 (8)	0.0036 (7)	0.0164 (8)
C17	0.0257 (8)	0.0539 (12)	0.0338 (10)	0.0032 (8)	0.0041 (7)	0.0269 (9)
C5	0.0448 (11)	0.0257 (9)	0.0312 (9)	0.0176 (8)	0.0076 (8)	0.0102 (7)
C15	0.0239 (7)	0.0238 (8)	0.0144 (7)	0.0058 (6)	−0.0024 (6)	0.0076 (6)
F1A	0.0482 (12)	0.110 (4)	0.061 (2)	−0.0003 (16)	0.0112 (12)	0.065 (3)
F2A	0.0699 (18)	0.0644 (15)	0.0221 (12)	0.0077 (14)	−0.0076 (11)	0.0157 (10)
F3A	0.152 (5)	0.079 (2)	0.0424 (16)	0.088 (3)	0.032 (2)	0.0407 (16)
F1B	0.246 (19)	0.101 (8)	0.030 (3)	0.134 (11)	0.048 (7)	0.039 (4)
F2B	0.030 (3)	0.172 (11)	0.080 (6)	0.022 (4)	0.001 (3)	0.102 (7)
F3B	0.082 (5)	0.130 (10)	0.068 (5)	−0.056 (6)	−0.024 (4)	0.078 (6)
F4A	0.064 (4)	0.047 (3)	0.0371 (13)	0.041 (3)	−0.0028 (15)	0.0078 (14)
F5A	0.038 (3)	0.038 (3)	0.0217 (16)	0.015 (2)	0.0063 (17)	0.0102 (15)
F6A	0.0388 (19)	0.0193 (12)	0.0317 (15)	0.0012 (11)	0.0010 (11)	0.0006 (9)
F4B	0.054 (4)	0.061 (5)	0.039 (2)	0.042 (4)	0.014 (2)	0.022 (3)
F5B	0.038 (5)	0.030 (3)	0.048 (7)	0.000 (3)	0.019 (4)	0.006 (3)
F6B	0.070 (6)	0.0170 (18)	0.072 (5)	−0.001 (2)	0.028 (5)	−0.004 (2)
F7	0.0507 (8)	0.0669 (10)	0.1391 (16)	−0.0257 (7)	−0.0618 (10)	0.0715 (11)
F8	0.0389 (7)	0.0730 (9)	0.0362 (6)	−0.0255 (6)	−0.0048 (5)	0.0285 (6)
F9	0.0531 (8)	0.0427 (7)	0.0511 (8)	−0.0181 (6)	0.0182 (6)	−0.0156 (6)
F10	0.0575 (7)	0.0416 (7)	0.0393 (6)	−0.0161 (6)	−0.0194 (5)	0.0301 (6)
F11	0.0288 (6)	0.0768 (9)	0.0450 (7)	0.0097 (6)	−0.0045 (5)	0.0380 (7)
F12	0.0936 (10)	0.0337 (6)	0.0180 (5)	0.0232 (6)	−0.0031 (6)	0.0060 (5)

### Geometric parameters (Å, º)

**Table:**

Co1—O3	2.0549 (11)	C3—H3	0.9300
Co1—O4	2.0805 (11)	C19—C15	1.420 (2)
Co1—O2	2.0875 (11)	C10—F10	1.3182 (19)
Co1—O1	2.0906 (11)	C10—F11	1.325 (2)
Co1—N1	2.1183 (13)	C10—F12	1.3315 (19)
Co1—N2	2.1318 (14)	C11—C12	1.408 (2)
O1—C1	1.2487 (19)	C11—H11	0.9300
O2—C4	1.2515 (19)	C4—C5	1.538 (2)
O3—C6	1.2500 (19)	C14—C16	1.411 (3)
O4—C9	1.2529 (19)	C14—C15	1.415 (2)
N1—C11	1.323 (2)	C7—F7	1.303 (2)
N1—C15	1.369 (2)	C7—F8	1.316 (2)
N2—C19	1.446 (2)	C7—F9	1.323 (2)
N2—H2A	0.9000	C2—F1B	1.215 (7)
N2—H2B	0.9000	C2—F3A	1.284 (4)
C18—C19	1.368 (2)	C2—F2B	1.285 (9)
C18—C17	1.412 (3)	C2—F1A	1.296 (3)
C18—H18	0.9300	C2—F2A	1.367 (4)
C8—C9	1.394 (2)	C2—F3B	1.417 (8)
C8—C6	1.399 (2)	C12—H12	0.9300
C8—H8	0.9300	C16—C17	1.365 (3)
C13—C12	1.358 (3)	C16—H16	0.9300
C13—C14	1.415 (3)	C17—H17	0.9300
C13—H13	0.9300	C5—F6B	1.202 (8)
C6—C7	1.539 (2)	C5—F5B	1.271 (18)
C1—C3	1.396 (2)	C5—F4A	1.293 (4)
C1—C2	1.541 (2)	C5—F5A	1.346 (13)
C9—C10	1.543 (2)	C5—F6A	1.427 (5)
C3—C4	1.391 (2)	C5—F4B	1.445 (10)
			
O3—Co1—O4	88.59 (4)	O2—C4—C3	128.69 (15)
O3—Co1—O2	92.89 (5)	O2—C4—C5	113.84 (14)
O4—Co1—O2	86.96 (4)	C3—C4—C5	117.38 (15)
O3—Co1—O1	91.57 (5)	C16—C14—C13	123.78 (16)
O4—Co1—O1	173.56 (4)	C16—C14—C15	118.94 (16)
O2—Co1—O1	86.60 (4)	C13—C14—C15	117.26 (16)
O3—Co1—N1	92.56 (5)	F7—C7—F8	107.38 (16)
O4—Co1—N1	93.50 (5)	F7—C7—F9	107.93 (17)
O2—Co1—N1	174.54 (5)	F8—C7—F9	106.06 (15)
O1—Co1—N1	92.92 (5)	F7—C7—C6	111.98 (14)
O3—Co1—N2	171.64 (5)	F8—C7—C6	113.86 (14)
O4—Co1—N2	93.30 (5)	F9—C7—C6	109.31 (14)
O2—Co1—N2	95.34 (5)	F1B—C2—F3A	122.2 (5)
O1—Co1—N2	87.47 (5)	F1B—C2—F2B	113.4 (8)
N1—Co1—N2	79.21 (5)	F1B—C2—F1A	65.7 (9)
C1—O1—Co1	126.51 (11)	F3A—C2—F1A	112.9 (3)
C4—O2—Co1	127.00 (11)	F2B—C2—F1A	127.6 (5)
C6—O3—Co1	125.51 (10)	F3A—C2—F2A	103.7 (3)
C9—O4—Co1	124.61 (10)	F2B—C2—F2A	86.9 (6)
C11—N1—C15	118.09 (14)	F1A—C2—F2A	104.3 (3)
C11—N1—Co1	128.77 (12)	F1B—C2—F3B	108.0 (7)
C15—N1—Co1	112.72 (10)	F3A—C2—F3B	75.6 (6)
C19—N2—Co1	109.49 (10)	F2B—C2—F3B	94.7 (7)
C19—N2—H2A	109.8	F1A—C2—F3B	46.1 (6)
Co1—N2—H2A	109.8	F2A—C2—F3B	141.1 (4)
C19—N2—H2B	109.8	F1B—C2—C1	118.3 (3)
Co1—N2—H2B	109.8	F3A—C2—C1	115.0 (2)
H2A—N2—H2B	108.2	F2B—C2—C1	112.9 (5)
C19—C18—C17	120.25 (17)	F1A—C2—C1	111.35 (18)
C19—C18—H18	119.9	F2A—C2—C1	108.60 (19)
C17—C18—H18	119.9	F3B—C2—C1	106.4 (3)
C9—C8—C6	122.01 (15)	C13—C12—C11	119.11 (16)
C9—C8—H8	119.0	C13—C12—H12	120.4
C6—C8—H8	119.0	C11—C12—H12	120.4
C12—C13—C14	119.87 (15)	C17—C16—C14	120.24 (17)
C12—C13—H13	120.1	C17—C16—H16	119.9
C14—C13—H13	120.1	C14—C16—H16	119.9
O3—C6—C8	129.11 (15)	C16—C17—C18	120.95 (17)
O3—C6—C7	113.78 (13)	C16—C17—H17	119.5
C8—C6—C7	117.03 (14)	C18—C17—H17	119.5
O1—C1—C3	129.16 (15)	F6B—C5—F5B	118.2 (12)
O1—C1—C2	113.85 (15)	F6B—C5—F4A	78.3 (6)
C3—C1—C2	116.98 (15)	F5B—C5—F4A	110.8 (13)
O4—C9—C8	129.33 (14)	F6B—C5—F5A	113.3 (10)
O4—C9—C10	113.31 (13)	F4A—C5—F5A	118.9 (8)
C8—C9—C10	117.35 (14)	F5B—C5—F6A	105.6 (9)
C4—C3—C1	121.85 (15)	F4A—C5—F6A	103.5 (4)
C4—C3—H3	119.1	F5A—C5—F6A	98.0 (6)
C1—C3—H3	119.1	F6B—C5—F4B	108.7 (5)
C18—C19—C15	119.70 (16)	F5B—C5—F4B	88.6 (12)
C18—C19—N2	123.99 (15)	F5A—C5—F4B	98.1 (7)
C15—C19—N2	116.28 (14)	F6A—C5—F4B	132.8 (5)
F10—C10—F11	107.19 (14)	F6B—C5—C4	115.5 (4)
F10—C10—F12	107.37 (14)	F5B—C5—C4	113.9 (9)
F11—C10—F12	106.61 (14)	F4A—C5—C4	115.3 (2)
F10—C10—C9	114.31 (13)	F5A—C5—C4	111.8 (6)
F11—C10—C9	110.84 (13)	F6A—C5—C4	106.6 (3)
F12—C10—C9	110.16 (13)	F4B—C5—C4	107.9 (4)
N1—C11—C12	123.29 (16)	N1—C15—C14	122.25 (15)
N1—C11—H11	118.4	N1—C15—C19	117.98 (14)
C12—C11—H11	118.4	C14—C15—C19	119.76 (15)
			
O3—Co1—O1—C1	−93.11 (14)	Co1—O2—C4—C5	179.71 (11)
O2—Co1—O1—C1	−0.30 (13)	C1—C3—C4—O2	0.7 (3)
N1—Co1—O1—C1	174.25 (14)	C1—C3—C4—C5	−175.65 (16)
N2—Co1—O1—C1	95.20 (14)	C12—C13—C14—C16	177.71 (16)
O3—Co1—O2—C4	88.38 (13)	C12—C13—C14—C15	−1.2 (2)
O4—Co1—O2—C4	176.82 (13)	O3—C6—C7—F7	36.9 (2)
O1—Co1—O2—C4	−3.02 (13)	C8—C6—C7—F7	−145.89 (17)
N2—Co1—O2—C4	−90.15 (14)	O3—C6—C7—F8	159.01 (15)
O4—Co1—O3—C6	9.12 (12)	C8—C6—C7—F8	−23.8 (2)
O2—Co1—O3—C6	96.00 (12)	O3—C6—C7—F9	−82.60 (18)
O1—Co1—O3—C6	−177.32 (12)	C8—C6—C7—F9	94.57 (18)
N1—Co1—O3—C6	−84.33 (12)	O1—C1—C2—F1B	−17.8 (13)
O3—Co1—O4—C9	−8.77 (12)	C3—C1—C2—F1B	163.1 (13)
O2—Co1—O4—C9	−101.74 (12)	O1—C1—C2—F3A	−174.6 (4)
N1—Co1—O4—C9	83.71 (12)	C3—C1—C2—F3A	6.3 (4)
N2—Co1—O4—C9	163.08 (12)	O1—C1—C2—F2B	−153.5 (7)
O3—Co1—N1—C11	8.91 (13)	C3—C1—C2—F2B	27.4 (7)
O4—Co1—N1—C11	−79.83 (13)	O1—C1—C2—F1A	55.3 (4)
O1—Co1—N1—C11	100.61 (13)	C3—C1—C2—F1A	−123.8 (3)
N2—Co1—N1—C11	−172.53 (14)	O1—C1—C2—F2A	−59.0 (3)
O3—Co1—N1—C15	−163.40 (10)	C3—C1—C2—F2A	121.9 (2)
O4—Co1—N1—C15	107.86 (10)	O1—C1—C2—F3B	103.9 (8)
O1—Co1—N1—C15	−71.70 (10)	C3—C1—C2—F3B	−75.2 (8)
N2—Co1—N1—C15	15.16 (10)	C14—C13—C12—C11	−1.6 (2)
O4—Co1—N2—C19	−111.13 (10)	N1—C11—C12—C13	2.2 (2)
O2—Co1—N2—C19	161.62 (10)	C13—C14—C16—C17	−179.35 (16)
O1—Co1—N2—C19	75.27 (10)	C15—C14—C16—C17	−0.4 (2)
N1—Co1—N2—C19	−18.19 (10)	C14—C16—C17—C18	−2.2 (3)
Co1—O3—C6—C8	−6.4 (2)	C19—C18—C17—C16	1.4 (3)
Co1—O3—C6—C7	170.37 (10)	O2—C4—C5—F6B	−95.2 (13)
C9—C8—C6—O3	−0.8 (3)	C3—C4—C5—F6B	81.7 (13)
C9—C8—C6—C7	−177.45 (14)	O2—C4—C5—F5B	46.3 (10)
Co1—O1—C1—C3	3.9 (3)	C3—C4—C5—F5B	−136.8 (10)
Co1—O1—C1—C2	−175.04 (11)	O2—C4—C5—F4A	176.1 (8)
Co1—O4—C9—C8	5.7 (2)	C3—C4—C5—F4A	−7.1 (8)
Co1—O4—C9—C10	−175.38 (9)	O2—C4—C5—F5A	36.2 (6)
C6—C8—C9—O4	1.1 (3)	C3—C4—C5—F5A	−146.9 (6)
C6—C8—C9—C10	−177.80 (14)	O2—C4—C5—F6A	−69.7 (3)
O1—C1—C3—C4	−4.7 (3)	C3—C4—C5—F6A	107.2 (3)
C2—C1—C3—C4	174.23 (17)	O2—C4—C5—F4B	143.0 (7)
C17—C18—C19—C15	1.9 (2)	C3—C4—C5—F4B	−40.1 (7)
C17—C18—C19—N2	−176.04 (15)	C11—N1—C15—C14	−3.4 (2)
Co1—N2—C19—C18	−162.39 (13)	Co1—N1—C15—C14	169.82 (11)
Co1—N2—C19—C15	19.57 (16)	C11—N1—C15—C19	177.73 (14)
O4—C9—C10—F10	174.74 (14)	Co1—N1—C15—C19	−9.06 (16)
C8—C9—C10—F10	−6.2 (2)	C16—C14—C15—N1	−175.13 (14)
O4—C9—C10—F11	53.48 (18)	C13—C14—C15—N1	3.9 (2)
C8—C9—C10—F11	−127.44 (16)	C16—C14—C15—C19	3.7 (2)
O4—C9—C10—F12	−64.27 (18)	C13—C14—C15—C19	−177.28 (14)
C8—C9—C10—F12	114.81 (16)	C18—C19—C15—N1	174.41 (14)
C15—N1—C11—C12	0.3 (2)	N2—C19—C15—N1	−7.5 (2)
Co1—N1—C11—C12	−171.68 (12)	C18—C19—C15—C14	−4.5 (2)
Co1—O2—C4—C3	3.3 (3)	N2—C19—C15—C14	173.64 (13)

### Hydrogen-bond geometry (Å, º)

**Table:**

*D*—H···*A*	*D*—H	H···*A*	*D*···*A*	*D*—H···*A*
N2—H2*A*···O2^i^	0.90	2.14	3.024 (2)	169
N2—H2*B*···O4^i^	0.90	2.56	3.069 (2)	117

Symmetry code: (i) −*x*, −*y*, −*z*+1.
